# *Skeletal muscle*’s 3rd year anniversary

**DOI:** 10.1186/2044-5040-4-3

**Published:** 2014-01-24

**Authors:** David J Glass, Kevin P Campbell, Michael A Rudnicki

**Affiliations:** 1 Novartis Institutes for Biomedical Research, Cambridge, MA, USA; 2 Howard Hughes Medical Institute, Molecular Physiology and Biophysics, Carver College of Medicine, University of Iowa, Iowa City, IA, USA; 3 Ottawa Hospital Research Institute and University of Ottawa, Ottawa, ON, Canada

## 

Three years ago *Skeletal Muscle* was launched to serve scientists who study the mechanistic basis of skeletal muscle development, homeostasis and disease. At the journal’s inception, we pointed out that cellular signaling and molecular genetics had combined to allow for a tremendous increase in the understanding of the fundamental processes that are distinct to skeletal muscle, in addition to ubiquitious signaling pathways which have particular effects or import on this tissue [1]. This influx of data seemed to more than justify a journal that could provide a home for this work. The point we made three years ago is even more justified today. Novel mechanisms have been published around many aspects of basic skeletal muscle biology, and this journal has featured quite a few of these discoveries.

Indeed, it was quite gratifying to see important work published quite early in *Skeletal Muscle*’s lifetime, papers which were rapidly downloaded thousands of times. That’s one big advantage of this journal - as an Open Access instrument, scientists can see their work disseminated widely, to any scientist, student or layperson who finds the work of interest, without any barrier or firewall. We’ve tried to make sure the journal maintains a very high standard of work, avoiding review of purely descriptive or incompletely characterized findings, and making sure all articles are fully reviewed, statistically sound and from principled authors. Indeed, we feel all prospective authors would be pleased to know that their papers would be reviewed by experts in the field, often members of our Editorial Board.

And yet we’re just beginning. We thank the community for its enthusiastic support of the journal, and hope this continues and strengthens. Please let us know how we can do an even better job to present important results in this rapidly-evolving field, and please do consider *Skeletal Muscle* early as a high-profile setting for your manuscripts.
